# Survey of Medical Referral by Japanese Dentists for Patients With Hepatitis B, Hepatitis C, and Lichen Planus

**DOI:** 10.7759/cureus.60624

**Published:** 2024-05-19

**Authors:** Yumiko Nagao, Kiyohide Tomooka, Hirokazu Takahashi

**Affiliations:** 1 Department of Public Health, Graduate School of Medicine, Juntendo University, Tokyo, JPN; 2 Liver Center, Saga University Hospital, Saga, JPN

**Keywords:** hepatologist, dental clinic, lichen planus, hepatitis b virus, hepatitis c virus

## Abstract

Objectives: This study aimed to investigate the referral rates of oral lichen planus (OLP) and untreated hepatitis virus-infected patients by dentists to hepatologists.

Materials and methods: The study was conducted at three dental clinics in the Oita prefecture between November 2021 and June 2023. Two distinct groups of patients who visited the dentist for dental treatment were included: those with liver disease and concurrent hepatitis C virus (HCV) or hepatitis B virus (HBV) infection and those diagnosed with OLP. The rate of medical referrals to a hepatologist was investigated. Data on the number of patients, gender, age, diagnosis of liver disease, and referral practices were collected from the records submitted by each dental clinic. Information about the HCV and HBV infection status was collected through interviews with the dentists.

Results: A total of 1,665 patients were included, of which 10 were HCV-infected, five were HBV-infected, and six were diagnosed with OLP. None of the 15 patients with liver disease were referred to a hepatologist by their dentists. Nine out of the 10 HCV-infected patients had achieved sustained virological response (SVR) after antiviral treatment. Of the six patients with OLP, one had a history of HBV infection, one had severe fatty liver, and the remaining four had normal livers; five of the OLP patients were referred to a hepatologist (83.3%).

Conclusion: A high referral rate from dentists to hepatologists was observed among the OLP patients. However, the study highlighted the difficulties in identifying hepatitis patients and establishing appropriate medical coordination in dental institutions.

## Introduction

Nosocomial infection control in dental institutions is an important issue for dental healthcare workers who are frequently in contact with the blood, saliva, and bodily fluids of patients [1]. When patients infected with hepatitis C virus (HCV) or hepatitis B virus (HBV) visit a dental clinic, dental healthcare workers are required to have sufficient knowledge about hepatitis viruses and must contribute to providing the required treatment along with medical care [2].

Lichen planus, a chronic inflammatory disease affecting both oral and cutaneous regions, is associated with HCV infection [3]. Several meta-analyses have reported a strong association between HCV and lichen planus [4-7] and shown that the successful elimination of HCV contributes to the healing of lichen planus [8-9]. Direct-acting antivirals (DAAs) can effectively treat hepatitis C, reduce total mortality [10], and decrease the risk of death from extrahepatic manifestations [11].

Advances in antiviral therapy have led to a decrease in liver cancer caused by viral liver disease in Japan [12]. However, there are an estimated 1.91-2.49 million HCV- and HBV-infected individuals in Japan, many of whom are unaware of their infection [13]. While it remains difficult for untreated HCV- and HBV-infected patients to receive adequate treatment, early detection of potential hepatitis patients and appropriate connection to treatment remain an important challenge [14]. Therefore, as healthcare providers, dentists also need to refer suspected hepatitis patients to hepatologists. We have previously reported on the identification of hepatitis patients by dentists in several regions in Japan [15-17]; oral lichen planus (OLP) patients must have access to medical care from a hepatologist during the consultation process.

In the Oita prefecture, workplace health examinations for hepatitis were conducted for dental healthcare workers in 2018 and 2022 [18-19], during which they were tested for hepatitis B surface antigen (HBsAg), antibodies to HBsAg (anti-HBs), and antibodies to hepatitis C virus (anti-HCV). Additionally, hepatitis education was provided using a hepatitis booklet in 2021 [20]. Therefore, the current study was conducted in this prefecture to assess the current situation. Comparisons of the liver cancer mortality rates per 100,000 individuals among the various prefectures in Japan revealed that the Oita prefecture ranked seventh in 2021 (mortality rate of 26.7%) [21] and fourth in 2022 (mortality rate of 26.8%) [22]. This study aimed to investigate whether dentists can refer untreated hepatitis virus-infected or OLP patients to a hepatologist for examination or consultation during the dental visit, thereby demonstrating the role of dentists in promoting appropriate medical coordination.

The Japanese healthcare system differs from that of many Western countries. In Western countries, there is often a clear referral pathway from dentists to oral medicine, oral and maxillofacial surgery, and dermatology for patients with OLP, whereas in Japan, general dentists play a more central role in patient management. The referral processes vary, and cooperation between dental and medical specialists is essential to ensure appropriate patient care.

## Materials and methods

Study design

The study was conducted in the Oita prefecture from November 17, 2021, to June 30, 2023. The data collection period at each dental clinic was 90 days. Dental clinics were invited to participate in the study through a call organized by the board of directors of the Oita Dental Association. Three dental clinics consented to the study, one of which was a facility with a full-time oral surgeon. This study focused on the collaboration between dentists and hepatologists, with a specific interest in patients with liver disease, HCV or HBV infection, and OLP.

Patients who visited these clinics for dental care were included in the study if they met the following criteria: having liver disease, HCV or HBV infection, or a clinical or clinical-pathological diagnosis of OLP. The Japanese insurance system does not permit blood tests on outpatients receiving dental care to determine the presence of HCV or HBV infection. In the study, the presence of HCV and HBV infection was assessed mainly through patient interviews. During these interviews, patients were asked about their medical history, including any prior diagnosis of hepatitis or liver disease, recent treatment of hepatitis, and if they had undergone treatment for HCV or HBV. This information was then used to categorize patients and determine their eligibility for the study. By focusing on interview-based assessments, we ensured a non-invasive approach that respected patient confidentiality and adhered to the Japanese insurance system's regulations. A dedicated referral form was used to refer the liver disease and OLP patients to hepatologists.

Data collection

Data collection involved obtaining information from each of the three dental clinics in electronic format, primarily using Excel spreadsheets. The data included the total number of patients, gender, age, diagnosis of liver disease, and whether the patient was referred to a hepatologist. These electronic data files were submitted by the directors of each dental clinic, ensuring data consistency and compliance with ethical guidelines. The data was anonymized to maintain patient confidentiality.

Hepatologists performed blood biochemistry tests and measured the hepatitis virus markers, such as anti-HCV and HBsAg. Additionally, abdominal imaging tests, including abdominal echo- or elastography (FibroScan; ECHOSENS, Paris, France), were conducted on each patient. The liver disease was diagnosed based on the results of these tests. A key focus of the study was documenting patient referrals to hepatologists, using standardized referral forms to ensure consistency across different dental clinics.

The data collection process was designed to maintain accuracy and reliability. Each dental clinic followed a standardized protocol for collecting and submitting data.

Collected data

The following data were collected and analyzed during the study: (a) total number of patients who visited the dental clinics during the data collection period, (b) number of HCV-infected patients, with gender, average age, and diagnosis of liver disease, (c) number of HBV-infected patients, with gender, average age, and diagnosis of liver disease, (d) number of OLP patients, with gender, average age, and diagnosis of liver disease, and (e) number of referrals from dentists to hepatologists for each category, along with the outcome of these referrals.

Data analysis

Although formal statistical analysis was not conducted due to the small sample size, the collected data was analyzed to identify trends and assess the collaboration between dentists and hepatologists. The analysis focused on determining the frequency and outcomes of referrals, the patterns of liver disease diagnoses, and the collaboration between different healthcare professionals.

Ethics approval statement

The study protocol was approved by the Ethics Committee of Saga University (reference number: R4-33) and Juntendo University (reference number: 2020259) in accordance with the Declaration of Helsinki. Informed consent was obtained from all participants prior to participation in the study. Although written consent was not obtained from each patient individually, posters were displayed in participating dental clinics as a means of informing patients about the study and providing them with the opportunity to refuse participation if they so desired. The chief dentists in these dental clinics understood the purpose and procedures of the study and gave their written consent for their dental clinics to participate in the study. Patients were assured that their decision to participate or not would not affect their treatment at the practice. Patient confidentiality and privacy were strictly maintained throughout the study, and all data were anonymized to ensure anonymity.

## Results

Of the 1,665 patients included in this study, 10 were infected with HCV and five with HBV (Table 1).

**Table 1:** Characteristics of subjects in this study HCV: hepatitis C virus; HBV: hepatitis B virus

Category	Number of patients (%)	Sex (male/female)	Average age (years)	Referral from dentist to physician (%)
HCV-infected patients	10 (0.6%)	2/8	70.6	0 (0%)
HBV-infected patients	5 (0.3%)	3/2	65.0	0 (0%)
Oral lichen planus	6 (0.4%)	2/4	69.3	5 (83.3%)

Among the HCV-infected patients, one was diagnosed with chronic hepatitis C, and nine presented with sustained virological response (SVR) after antiviral therapy (Table 2).

**Table 2:** Diagnosis of liver disease in patients with viral infections and oral lichen planus HCV: hepatitis C virus; HBV: hepatitis B virus; CH-C: chronic hepatitis C; SVR: sustained virological response; CH-B: chronic hepatitis B; LC-B: HBV-related liver cirrhosis; HCC: hepatocellular carcinoma

Category (n)	Liver disease	Number
HCV infection (10)	CH-C	1
	CH-C SVR	9
HBV infection (5)	CH-B	4
	LC-B+post HBV-related HCC	1
Oral lichen planus (6)	Past HBV infection	1
	Severe fatty liver	1
	Normal liver	4

Among the HBV-infected patients, four were diagnosed with chronic hepatitis B and one was diagnosed with cirrhosis type B after post-treatment hepatocellular carcinoma. None of these 15 patients with liver disease were referred to a hepatologist by their dentist.

Six patients were diagnosed with OLP, and five (83.3%) of them were referred to hepatologists by the dentist using a dedicated referral form (Table 1). Among the OLP patients, one was diagnosed with liver disease and a history of HBV infection, one was diagnosed with severe fatty liver, and four were diagnosed with no liver abnormalities.

## Discussion

The results of this study indicate that the profile of hepatitis virus-infected patients who receive dental care has changed compared to that in previous studies, where the majority of hepatitis C patients who visited the dentist were untreated and persistently infected [17]. In the present study, nine out of 10 HCV-infected patients who visited the dentist had already achieved SVR with antiviral treatment, reflecting the recent advances in treatment. This change may be attributed to the widespread use of DAA therapy, which has shortened the duration of hepatitis C treatment and reduced its side effects. In this study, patients self-reported that they had achieved SVR, and the dentists might not have deemed it necessary to refer them to a hepatologist.　

However, not all patients with liver disease were referred to a hepatologist in this study, thus highlighting the challenges dentists face in recommending examinations and treatments for liver diseases to other physicians. Dentists focus mainly on providing dental care for their patients; their expertise and ability to provide appropriate treatments for liver disease are limited. Therefore, collaborations between dentists and physicians are essential, and the dentist must be able to refer patients with liver disease to appropriate medical facilities.

It is noteworthy that the recommendation rate for consultation for liver disease among OLP patients was as high as 83.3%, presumably because it is easier for dentists to explain to patients that OLP is associated with liver disease and advise them to undergo treatment for the disease. Recently, periodontal pathogens have been reported to be associated with the development of nonalcoholic fatty liver disease (NAFLD) and nonalcoholic steatohepatitis (NASH) [23], both of which can progress to liver cancer [24]. Therefore, treatment of periodontal disease may be an important aspect in preventing and managing lifestyle-related diseases. The incidence of NAFLD and NASH can be reduced by improving the oral environment and treating periodontal disease [25]. These findings indicate that it is important for dentists to refer OLP patients and those with periodontal diseases to appropriate medical facilities, as required.

HCV-infected patients have a high complication rate of OLP [26-27]. Younossi et al. conducted a meta-analysis of the prevalence of extrahepatic manifestations, including the prevalence and consequences of lichen planus, in HCV-infected patients in East Asia [26]. They found that the prevalence of lichen planus was 8.9% in HCV-infected patients in East Asia (Japan, China, and Taiwan) and 4.2% in HCV-uninfected patients. Furthermore, HCV-infected patients had a significantly higher risk of developing lichen planus (odds ratio, 2.21). Younossi et al. performed another meta-analysis of the prevalence of extrahepatic manifestations in HCV-infected patients in Western countries and reported that the risk in HCV-infected patients was more than twice that in uninfected patients 1.9% in HCV-infected patients and 1.1% in HCV-uninfected patients [27].

In the present study, 0.6% (10/1,665) of the patients were HCV-infected, and 0.3% (5/1,665) were HBV-infected, based on the results of the medical interviews. However, given the high liver cancer mortality rate in the Oita prefecture [21-22], the number of potentially infected patients must be high in the population. In one study comprising 227 patients who visited the dentist and were determined via interviews to have no history or risk of HBV and HCV infection (history of blood transfusion, dialysis, history of liver disease, family history of liver disease), the actual anti-HCV antibody positivity rate was reported to be 3.5% (8/227 patients) [28]. In addition, an HBV and HCV infection survey of 703 patients who underwent outpatient dental and oral surgery procedures reported that only 16.7% of the 18 infected patients were identified via interviews [29]. These findings indicate the likelihood of encountering patients with hepatitis virus infection among those who visit the dental clinic despite the absence of a history or risk of hepatitis. Furthermore, these results indicate the limitations of the medical interview method and the insurance system for dental care in Japan.

General dentists are faced with several challenges while dealing with hepatitis patients. First is the lack of expertise regarding the disease. Dentists are oral specialists and need specialized education and information to acquire knowledge on hepatitis. Secondly, they lack access to academic information and the latest medical knowledge on hepatitis. Thirdly, the lack of motivation may make it difficult to attract their interest in learning about hepatitis because it is not directly related to their reimbursement. A lack of support in providing information on hepatitis to dentists limits their access to the latest information. Thus, providing professional education and information to dentists must be improved to overcome these challenges.

This study has some limitations. First, it was based on data from three dental clinics in the Oita prefecture. The small number of surveyed medical institutions may have influenced the results of this study. Second, the study period coincided with the coronavirus disease 2019 (COVID-19) pandemic, when patients were less likely to have visited the dentist. Collecting data from a wider, more diverse range of medical institutions is desirable. The difference in healthcare systems between Japan and Western countries can impact patient outcomes. While in Western countries, the referral process for patients with OLP typically involves specialized departments, in Japan, general dentists play a central role in patient care. This unique arrangement may lead to variations in patient referrals and treatment approaches, highlighting the importance of a more collaborative approach. To address these limitations and further investigate the implications of the study findings, future research could focus on evaluating the effectiveness of a collaborative treatment model involving dentists and hepatologists in improving patient outcomes. In addition, investigating barriers to referral and treatment of liver disease in the dental setting and identifying strategies to overcome these barriers may provide valuable insights to enhance patient care.

## Conclusions

This study revealed that general dentists tended to actively refer OLP patients to hepatologists, suggesting that it is relatively easy to scrutinize liver disease through OLP patients. On the other hand, the study highlighted the challenges dental institutions face in identifying hepatitis patients and establishing appropriate medical linkages. Continuous hepatitis education and support for dentists and reforming the insurance and healthcare systems will be necessary to address the challenges. A more comprehensive approach to data collection and disease management from a more diverse range of dental providers is warranted in the future.
